# Barriers and Facilitators to Delivering Multifactorial Risk Assessment and Communication for Personalized Breast Cancer Screening: A Qualitative Study Exploring Implementation in Canada

**DOI:** 10.3390/curroncol32030155

**Published:** 2025-03-10

**Authors:** Meghan J. Walker, Anna Neely, Antonis C. Antoniou, Mireille J. M. Broeders, Jennifer D. Brooks, Tim Carver, Jocelyne Chiquette, Douglas F. Easton, Andrea Eisen, Laurence Eloy, D. Gareth R. Evans, Samantha Fienberg, Yann Joly, Raymond H. Kim, Bartha M. Knoppers, Aisha K. Lofters, Hermann Nabi, Nora Pashayan, Tracy L. Stockley, Michel Dorval, Jacques Simard, Anna M. Chiarelli

**Affiliations:** 1 Ontario Health (Cancer Care Ontario), Toronto, ON M5G 2L3, Canada; anna.neely@ontariohealth.ca (A.N.); andrea.eisen@sunnybrook.ca (A.E.); samantha.fienberg@ontariohealth.ca (S.F.); raymond.kim@uhn.ca (R.H.K.); anna.chiarelli@ontariohealth.ca (A.M.C.); 2 Dalla Lana School of Public Health, University of Toronto, Toronto, ON M5S 1A1, Canada; jennifer.brooks@utoronto.ca (J.D.B.); aisha.lofters@utoronto.ca (A.K.L.); 3 Centre for Cancer Genetic Epidemiology, Department of Public Health and Primary Care, School of Clinical Medicine, University of Cambridge, Cambridge CB1 8RN, UK; aca20@medschl.cam.ac.uk (A.C.A.); tjc29@medschl.cam.ac.uk (T.C.); dfe20@medschl.cam.ac.uk (D.F.E.); np275@medschl.cam.ac.uk (N.P.); 4 IQ Health Science Department, Radboud University Medical Center, 6525 EP Nijmegen, The Netherlands; mireille.broeders@radboudumc.nl; 5 CHU de Québec-Université Laval Research Center, Québec City, QC G1V 4G2, Canada; jocelyne.chiquette@crchudequebec.ulaval.ca (J.C.); hermann.nabi@fmed.ulaval.ca (H.N.); michel.dorval@crchudequebec.ulaval.ca (M.D.); 6 Department of Family Medicine and Emergency Medicine, Faculty of Medicine, Université Laval, Quebec City, QC G1V 0A6, Canada; 7 Sunnybrook Health Science Center, Toronto, ON M4N 3M5, Canada; 8 Programme Québécois de Cancérologie, Ministère de la Santé et des Services Sociaux, Quebec City, QC G1S 2M1, Canada; laurence@laurenceeloy.com; 9 Department of Medical Genetics and Cancer Epidemiology, The University of Manchester, Manchester M13 9PL, UK; gareth.d.evans@manchester.ac.uk; 10 Centre of Genomics and Policy, McGill University, Montreal, QC H3A 0G1, Canada; yann.joly@mcgill.ca (Y.J.); bartha.knoppers@mcgill.ca (B.M.K.); 11 Princess Margaret Cancer Centre, Toronto, ON M5G 2M9, Canada; 12 Women’s College Research Institute, Toronto, ON M5G 1N8, Canada; 13 Department of Social and Preventive Medicine, Faculty of Medicine, Université Laval, Quebec City, QC G1V 0A6, Canada; 14 Cancer Research Center, Université Laval, Quebec City, QC G1R 3S3, Canada; 15 Division of Clinical Laboratory Genetics, University Health Network, Toronto, ON M5G 2C4, Canada; tracy.stockley@uhn.ca; 16 Department of Laboratory Medicine and Pathobiology, University of Toronto, Toronto, ON M5S 1A8, Canada; 17 Faculty of Pharmacy, Université Laval, Quebec City, QC G1V 0A6, Canada; 18 Department of Molecular Medicine, Faculty of Medicine, Université Laval, Quebec City, QC G1V 4G2, Canada

**Keywords:** risk-stratification, breast cancer screening, personalized screening, implementation, barriers, qualitative research

## Abstract

Many jurisdictions are considering a shift to risk-stratified breast cancer screening; however, evidence on the feasibility of implementing it on a population scale is needed. We conducted a prospective cohort study in the PERSPECTIVE I&I project to produce evidence on risk-stratified breast screening and recruited 3753 participants to undergo multifactorial risk assessment from 2019–2021. This qualitative study explored the perspectives of study personnel on barriers and facilitators to delivering multifactorial risk assessment and risk communication. One focus group and three one-on-one interviews were conducted and a thematic analysis conducted which identified five themes: (1) barriers and facilitators to recruitment for multifactorial risk assessment, (2) barriers and facilitators to completion of the risk factor questionnaire, (3) additional resources required to implement multifactorial risk assessment, (4) the need for a person-centered approach, and (5) and risk literacy. While risk assessment and communication processes were successful overall, key barriers were identified including challenges with collecting comprehensive breast cancer risk factor information and limited resources to execute data collection and risk communication activities on a large scale. Risk assessment and communication processes will need to be optimized for large-scale implementation to ensure they are efficient but robust and person-centered.

## 1. Introduction

Breast cancer represents a major global cause of morbidity and mortality [1], and evidence demonstrates that breast screening is an effective method for reducing its burden [2,3,4,5,6]. Like most screened cancers, eligibility for breast screening is based primarily on age [7,8]. Age-based screening does not consider variability in breast cancer risk in the population, meaning that considerable proportions of people will be under- or over-screened if they are screened based on age alone. Personalized or risk-stratified screening is one of the strategies proposed as an alternative to screening models based only on age [9,10]. In a risk-stratified approach, the age at which screening begins and ends, as well as screening modalities and intervals, are determined by a person’s individual level of risk based on a comprehensive set of recognized risk factors.

While risk-stratified screening appears to improve the benefit-to-risk ratio and cost-effectiveness of breast screening [11,12], evidence on its clinical effectiveness is still forthcoming and a great deal of knowledge is required to understand the feasibility of implementing it on a population basis. Personalized Risk Assessment for Prevention and Early Detection of Breast Cancer: Integration and Implementation (PERSPECTIVE I&I) [13] is one of the projects in an international network of studies collectively generating the necessary evidence to support a shift of the breast screening paradigm from age-based to risk-based [14,15,16,17,18,19]. PERSPECTIVE I&I is generating the first evidence on the delivery of risk-stratified screening incorporating multifactorial risk assessment with polygenic risk scores within existing organized breast screening programs in Canada.

We reported findings from a PERSPECTIVE I&I study which described the participants who adopted multifactorial risk assessment and evaluated methods for collecting breast cancer risk factor information [20]. Several of our findings could not be fully explained. For example, much-greater-than-expected proportions of participants withdrew at the risk factor questionnaire stage and required verification of their risk factor information due to missing or unusual values. There were also challenges collecting breast density from mammogram reports. To contextualize and explain our quantitative findings, we conducted a qualitative study using an emergent explanatory sequential approach [21]. The aim of this study was to identify barriers to and facilitators of performing multifactorial breast cancer risk assessment and communicating breast cancer risk to participants. Only one other study has been conducted, a qualitative study from the BC-Predict study in England, which examined barriers and facilitators of risk-stratified breast screening from the perspective of personnel and health care providers who delivered it [22]. This evidence is needed to inform optimal implementation strategies for risk-based breast screening on a population scale.

## 2. Materials and Methods

### 2.1. Study Setting, Population, and Design

The overarching goal of PERSPECTIVE I&I is to improve breast cancer risk assessment and identify optimal approaches for implementing risk-based screening and prevention in Canada by executing four interrelated activities: (i) identify and validate novel moderate and high-risk breast cancer susceptibility genes via whole-exome sequencing to support development of a comprehensive multi-gene panel test; (ii) improve, validate, and adapt a risk prediction web tool for the Canadian context; (iii) develop and pilot a socio-ethical framework to support implementation of risk-based breast screening at the population level; and (iv) perform economic simulation modeling to optimize the implementation of risk-stratified breast cancer screening. Within the third activity, a large prospective cohort study was undertaken to generate evidence on the delivery of multifactorial breast cancer risk assessment with polygenic risk scores and risk-stratified breast cancer screening in the population setting. Detailed descriptions of the cohort study were previously published [13,20]. Briefly, females aged 40–69 in Ontario and Quebec, Canada who had a previous mammogram were invited to undergo multifactorial breast cancer risk assessment and receive personalized breast cancer screening and prevention recommendations from July 2019 to December 2021. Those with a personal history of breast, ovarian, or pancreatic cancer or mastectomy, who were at known high risk of breast cancer, or previously had genetic testing or counseling for breast cancer, were not eligible. Quebec participants were also required to have a regular primary care provider.

Multifactorial breast cancer risk assessment involves predicting an individual’s risk of developing breast cancer within a specified time taking into account established genetic and non-genetic risk factors. These may include high-, moderate-, and low-penetrance genetic variants, family history of breast and other cancers, breast density, history of benign breast disease, hormonal factors, anthropometric characteristics, and lifestyle factors. To facilitate multifactorial risk assessment for this study, eligible people were asked to complete a questionnaire at study entry to collect information on breast cancer risk factors, provide a saliva sample as a source of DNA for a clinical-grade Breast Cancer Genetic Risk Single Nucleotide Polymorphisms (SNPs) test for calculation of their polygenic risk score (PRS) [23,24] and consent to the collection of their most recent mammogram report to obtain mammographic density. The entry questionnaire collected the detailed breast cancer risk factor information required by the CanRisk prediction tool [25], based on the validated Breast and Ovarian Analysis of Disease Incidence and Carrier Estimation Algorithm (BOADICEA) model [23,24,26,27,28], including first- and second-degree family history of breast, ovarian, prostate, and pancreatic cancer, Ashkenazi Jewish ancestry, as well as hormonal (i.e., menstrual and reproductive history, exogenous hormone use), anthropometric (i.e., height and weight) factors, and lifestyle (i.e., alcohol use) factors. While CanRisk can incorporate the effects of rare pathogenic variants in moderate- and high-risk susceptibility genes, the presence of variants in these genes was treated as unknown in CanRisk in this study. This is because variants in these susceptibility genes were not tested for in the context of our study and individuals with a prior history of genetic counseling or testing were excluded. The entry questionnaire could be completed online, on paper (Ontario only), or over the telephone (Ontario only). To ensure the accuracy of risk prediction, study personnel contacted some participants by telephone to fill in missing risk factor data or verify unusual values observed for specific risk factor questions (e.g., age at menarche <8 years). The questionnaire data, PRS, and mammographic density were entered into the CanRisk tool to estimate each participant’s 10-year breast cancer risk. Risk estimates were then stratified into three risk levels using age-specific remaining lifetime risk thresholds for people aged 30–80 years (anchored at 30) [29]: average (<15% remaining lifetime risk), higher than average (15 to <25% remaining lifetime risk), or high risk (≥25% remaining lifetime risk).

Participants then received a personalized letter containing their risk level and risk-stratified screening action plan, which was either mailed (Ontario) or accessed through an online portal (Quebec). In Quebec, participants’ health care providers also received a letter disclosing their patient’s risk level. In Ontario, all participants estimated to be at high risk or who met criteria for the high-risk stream of the Ontario Breast Screening Program (OBSP) were offered a referral to the high-risk program and genetic counseling. In addition, any Ontario participants of average or higher-than-average risk who wished to discuss their risk level with a genetic counselor could request an appointment. In Quebec, a study nurse called participants who were estimated to be at high risk to disclose their risk level and discuss their screening action plan prior to the risk letter being available on the portal. Risk assessment was performed for 3753 participants (*n* = 2111 Ontario; *n* = 1642 Quebec) and risk communicated to 3714 as 39 participants opted not to have their risk level disclosed.

### 2.2. Recruitment and Data Collection

Research personnel directly involved in recruiting study participants for multifactorial risk assessment, or in risk factor data collection and verification or risk communication processes, were eligible to participate. Personnel were invited via email to participate in a focus group or one-on-one interview session. Interested personnel contacted the research team to arrange a time for the session.

A semi-structured topic guide was developed to administer to personnel participating in the focus group who were involved in recruitment, data collection (i.e., administering questionnaires or supporting participants with questionnaire completion, preparation, and mailing of saliva sample kits, obtaining mammogram reports, or abstracting mammographic density information), and risk factor verification activities (i.e., contacting participants to fill in missing information of verify unusual values). A semi-structured topic guide was developed to administer to clinical personnel participating in interviews who carried out risk communication activities. The questions contained within the two topic guides focused on the experiences of personnel in executing the study, as well as an exploration of barriers and facilitators to future provincial or national implementation of risk-based breast screening (File S1). Several health frameworks were used to develop the guides, including the Predisposing, Reinforcing, and Enabling Constructs in Educational Diagnosis and Evaluation–Policy, Regulatory, and Organizational Constructs in Educational and Environmental Development (PRECEDE–PROCEED) model [30] and RE-AIM, a frequently used implementation science framework for evaluation of public health interventions [31]. The guides were developed iteratively (AN, MW) and reviewed by senior members of the project team.

The focus group and interviews were conducted in English in August 2023 via the Microsoft Teams videoconferencing platform by a researcher with extensive experience conducting qualitative research and no prior relationship with the PERSPECTIVE I&I project or its personnel (AN). Interviewees were provided with a copy of the relevant topic guide at least two weeks prior to their scheduled focus group or interview. An interpreter participated in one interview to support the translation of dialogue between the Francophone interviewee and interviewer. Informed consent to participate was obtained in writing prior to the focus group or interview session or verbally at the outset of the session. The focus group and interview sessions were recorded with audio and video on Microsoft Teams and data were subsequently transcribed verbatim.

### 2.3. Data Analysis

To achieve anonymity, participants were assigned a unique participant number and all identifying information was removed from quotes and replaced with the participant numbers. All transcripts were imported into NVivo^®^ 14 to facilitate a thematic analysis [32]. Themes were generated inductively by the researchers through a rigorous process of data familiarization, coding, theme development, and revision. The initial analysis was completed by one researcher (AN) who met with a second researcher (MW) to discuss coding and thematic structure. Three cycles of refinements were made until the researchers achieved consensus on a final thematic structure. The researchers selected illustrative quotes collaboratively. Regarding member checking, participants who remained active members of the project at the time the analysis was completed were invited to review and provide feedback on the draft findings. Their feedback was used to further refine the selection of illustrative quotes and summary text for several themes.

## 3. Results

**Figure 1:**
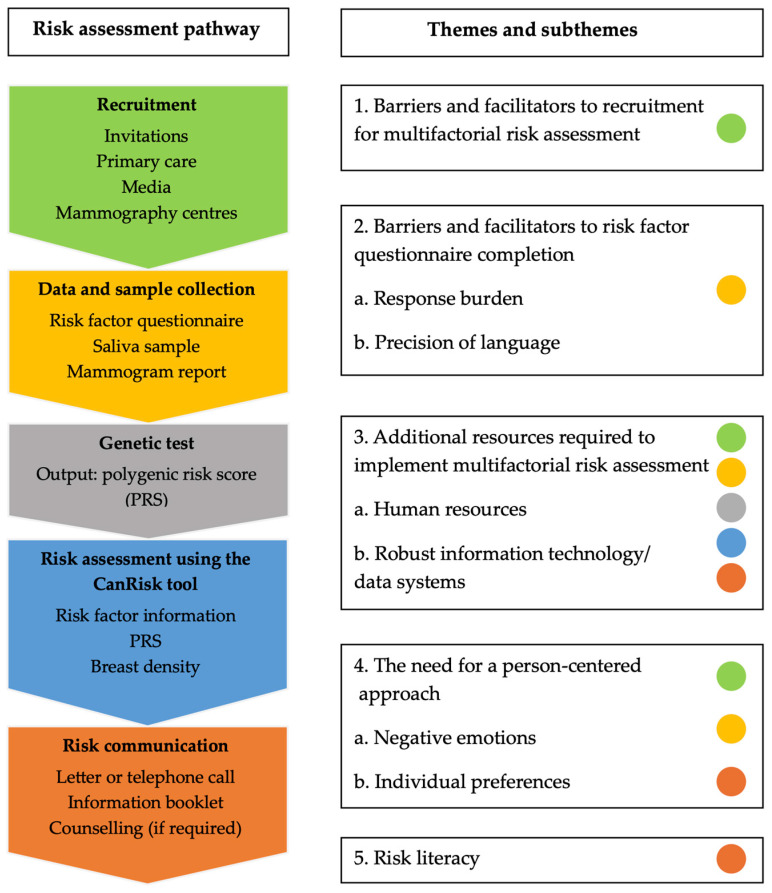
Themes and subthemes mapped to the multifactorial risk assessment pathway.

Nine PERSPECTIVE I&I personnel were invited and agreed to participate (response rate = 100%). The focus group was 90 min in duration and included six research personnel involved in recruitment, risk factor data collection, and verification. Three one-on-one interviews were completed with clinical personnel responsible for risk communication, with a mean duration of 50 min. Five themes and seven subthemes were identified, some which were relevant to single steps in the multifactorial risk assessment pathway and some of which emerged in relation to more than one step (Figure 1).

### 3.1. Theme 1: Barriers and Facilitators to Recruitment for Multifactorial Risk Assessment

Interviewees shared insights into the relative effectiveness of the various methods used to recruit participants to risk-stratified breast screening. In Ontario, recruitment strategies included mailed letters of invitation for people who had a previous mammogram at six OBSP sites, advertisements in mammography centers, and primary care providers, webpages, newsletters, and social media. In Quebec, recruitment was conducted though advertisement at mammography centers, traditional media, social media, email listservs of affiliate organizations, and a study website. The mailed invitation letters and traditional media (i.e., radio, television, and newspaper) campaigns, responsible for recruiting the vast majority of participants, were cited most frequently as being successful. Recruitment via social media experienced mixed success, and interviewees highlighted the limitations of social media’s ability to reach certain segments of the target population. Furthermore, interviewees commented on the multi-pronged approach to recruitment used by the study and the associated difficulty in determining which campaigns may have driven screening uptake within specific regions.

The COVID-19 pandemic was discussed as an external factor that hampered recruitment efforts throughout the study period; for example, public health restrictions posed a physical barrier to executing promotional activities at breast screening sites and recruitment through primary care providers was also challenged due to their focus on acute pandemic response and few patients being seen in person. Study eligibility criteria were also cited as an important factor that negatively affected recruitment, particularly with regards to the recruiting people aged 40–49 who were less likely to have had a previous mammogram. The requirement for Quebec participants to have a regular primary care provider or nurse practitioner represented a barrier for potential participants who were ‘unattached’ to a family physician or nurse practitioner.

It was recognized by interviewees that there would be an important role for primary healthcare providers in recruiting people to participate in future-state risk-based breast screening programs. Interviewees acknowledged that this approach would have limitations, as many Canadians currently do not have a regular primary care provider and many health care providers currently may lack knowledge about risk-based screening.

### 3.2. Theme 2: Barriers and Facilitators to Risk Factor Questionnaire Completion

#### 3.2.1. Response Burden

Interviewees reported that the study participants were often overwhelmed with the volume of information and the level of detail they were asked to provide in the risk factor questionnaire, particularly in relation to the family history section. This was felt to have negatively impacted some participants’ experience, the timeliness or accuracy of the risk assessment process, and in some cases, participation.

*“We could see at which point they [the participants] stopped in the questionnaire. Generally it was really around that family history [section] is where they stopped, because I think they were overwhelmed by that too…So they would know their first degree, mostly, but then when it came to second degree relatives, you know aunts and uncles and things like that, they sort of had a more difficult time around that.”*

(Participant 1)

*“But I think a good portion of it was really the family history section where we got a lot of inquiries about “how much information do I need?” and “if I don’t remember or don’t know my family history…” I think the part that sort of delayed some of the return of the questionnaires because individuals then want to go speak to their family members and get their family history… it was really the family history that seemed to cause, I think, issues around completion or around the questionnaire being fully completed and having that information.”*

(Participant 1)

#### 3.2.2. Precision of Language

The wording of certain questionnaire items, including questions regarding family history of breast cancer and hormonal risk factors, was also identified as a challenge to data collection for participants and was suspected to have often been the cause of both inaccurate and missing data:

*“People did not like answering that question. They were just like ‘this is ridiculous’, ‘This is just very confusing’. Again, I think it was just the language behind it…People are like ‘I’m not answering it, I’m confused by what you’re asking me’ and me even reading it, I’m confused by this question.”*

(Participant 6)

*“So specifically, the unilateral and the bilateral breast cancer question, I think a lot of the women were confused by the way the question was being asked.”*

(Participant 3)

#### 3.2.3. Digital Accessibility

Interviewees agreed that barriers to accessing the online questionnaire, including having the necessary equipment, skills, and knowledge to access the online platform, may have hindered people from accurately completing the questionnaire or from participating in multifactorial risk assessment altogether. Interviewees commented that even those who were able to access the online questionnaire sometimes struggled to complete it. Study team members observed that those who favored completing the questionnaire via paper or over the telephone instead of online were often older and/or were more likely to belong to a visible minority group, compared to the wider study cohort.

*“You would also need to think about, OK, so now…women are entering the questionnaire online or through a form. Is that a barrier for some women? Are we not catching a population of women if they’re first language is not English or they do not speak English at all, would that be an issue?”*

(Participant 8)

### 3.3. Theme 3: Additional Resources Required to Implement Multifactorial Risk Assessment

#### 3.3.1. Additional Human Resources

Interviewees repeatedly articulated the extensive time and efforts that were involved in contacting participants (primarily via telephone) for both risk factor data verification and risk communication. Numerous call attempts were typically required, often outside of standard office hours. When personnel were able to reach participants, phone calls were described as lasting up to an hour, occasionally longer, depending on the individual they were talking to and how many data points required verification:

*“So they [telephone calls for verifying risk factor data] were supposed to be 20 min. They were never 20 min. I would take a good 45 min to an hour…I’d rather spend an hour and get the right answers as opposed to rushing it in 20 min and you know it’s not filled out properly.”*

(Participant 6)

While data collection and data management processes surrounding saliva samples and mammogram reports were reported to have worked very smoothly for the most part, interviewees did highlight the extensive “legwork” or behind-the-scenes time and effort required for some tasks. For example, different processes for retrieving mammogram reports were required by different sites; some sites assumed the responsibility for locating and sending mammogram reports while others required study personnel to obtain direct access to their internal reporting systems to abstract reports themselves. Mammographic density was noted as being reported in different formats at different sites.

With regards to the processes surrounding the collection and processing of saliva samples, it was sometimes necessary for study team members to follow up with the receiving laboratory and/or resend saliva kits to participants that were assumed to have been lost in transit, as well as respond to queries from participants regarding saliva collection and DNA extraction.

*“So some of them [participants] say they were sick or they have a specific medical condition. So they were wondering if that could affect the sample. Or a lot of people were also not able to provide enough saliva…like “if I’m not able to provide enough, is it worth sending it or not?” Also, we received a lot of questions about the temperature because, you know…we have a lot of extreme temperatures…so they were again wondering if that could affect the integrity of the saliva sample.”*

(Participant 4)

Focus group and interview participants were asked to comment on the feasibility of scaling the processes used to collect data and communicate risk level to participants on a larger, provincial scale. Several challenges were identified, particularly surrounding the increase in volume of people being risk assessed and screened that would be associated with the implementation of a population-based risk-based breast screening program. The resources required to operationalize the collection and verification of self-reported risk information from screening participants was frequently mentioned, as were the costs of mailing saliva sample collection kits as well as the human resource capacity required to retrieve mammogram reports from a range of sources. Additionally, interviewees questioned the scalability of the resourcing required to effectively communicate risk and provide personalized follow-up information, referrals, and genetic counseling to screening participants identified as being ‘high-risk’.

*“If this was implemented at a population level…the energy that it takes to communicate this information [breast cancer risk] by phone…that would be a main challenge at a population level.”*

(Participant 7)

#### 3.3.2. Need for Robust Information Technology (IT) and Data Systems

Interviewees described the time and effort involved in managing several technical glitches that were detected via the study quality control processes. Study team members frequently described such occurrences as being “out of their control” as well as time-consuming and problematic. An example of one such glitch resulted in participants needing to be contacted to re-register for the study as increased traffic to the website resulted in their registration not processing. Another glitch also occurred with the use of an intermediate database used for risk factor information that had been corrected during the verification process, with interviewees highlighting the need for the centralization of all data within a single database to ensure data integrity and accuracy of risk estimates.

### 3.4. Theme 4: The Need for a Person-Centered Approach

#### 3.4.1. Negative Emotions

Interviewees who had direct communication with participants frequently commented on their perceived emotional state, both during risk factor data verification and risk communication conversations. While interviewees expressed that they felt most participants were not overtly emotive during their interactions, a minority were reported to have expressed some emotional distress or anxiety, more often among participants who received a ‘high-risk’ result.

Certain questions contained within the questionnaire were also found to trigger an emotional response for some participants, including those about previous pregnancies and the family history of cancer. Some participants who had been previously pregnant but not had a live birth, or had negative family relationships which impacted their ability to complete the family history, were reported to be distressed by the questions on these topics.

*“I found that some people were emotional…sometimes the person would say ‘I had kids’, but then they didn’t have the children. So it’s a very delicate situation… and in some cases too I found that some people didn’t have the information [family history] because they had negative family relationships…they would go on and on about how it’s been years since they’ve spoken to their family member, their siblings or their parents. So they had no information and they had no motivation to go get it because of that. So yeah, there was an emotional component… to some of those calls for sure…”*

(Participant 5)

#### 3.4.2. Individual Preferences for Data Collection and Risk Communication

The importance of tailoring both the data collection methods and risk communication to people’s individual preferences and needs was highlighted by interviewees. Some participants were reported to have experienced distress due to having their risk level communicated by letter instead of over the telephone or in person. Lastly, interviewees recognized the need to have a flexible model at the provincial scale that is person-centered, takes individuals’ preferences into account, and strives to mitigate equitable access concerns.

*“Some may prefer phone. Some may prefer in person. Others may prefer video and I think take home message is that perhaps it’s not one model fits all type of situation…especially for women who may not feel that comfortable using technology.”*

(Participant 8)

### 3.5. Theme 5: Risk Literacy

Risk literacy emerged as the final theme. Overall, interviewees described the study cohort as a group of health-motivated, information-seeking individuals. In addition to reviewing the individual’s risk level, risk communication conversations often included discussion of risk reduction measures, including lifestyle strategies, with conversations emphasizing how risk and risk reduction should be viewed as multifactorial:

*“I use the analogy of a Mason jar to describe women’s breast cancer risk. So it’s like a Mason jar and you have pebbles in the Mason jar. Each individual risk factor is a small or larger pebble. Some of them may contribute more, others less, but they collectively then give you kind of your risk level… people need to keep in mind…that their breast cancer risk is influenced by all these risk factors collectively and not an individual risk factor…the other thing is whether or not that’s manageable, actionable to them. There are things that we can control and there are things that we cannot control.”*

(Participant 8)

*“… a lot of questions regarding, you know, ‘if I change my lifestyle habits does my risk…improve?’…lifestyle habits are one component of the risk calculation, amongst other things. Lifestyle habits, if you change them, they’re good, of course, they’re good for anything else. But… it’s just one factor amongst others that are included in the risk.”*

(Participant 7)

Study team members reported that some participants expressed a desire for more precise information regarding their risk beyond the three-tiered risk level that was communicated:

*“So there was actually a question that came up a lot of times…within the high risk category, you know, ‘Am I at very high risk or am I at medium-high risk or am I at low-high risk?’. So that was… a preoccupation that came across various times.”*

(Participant 7)

*“ She [study participant] received an ‘average risk’ result and she actually was in distress about the information because she thought that she should really receive a ‘lower-than-average risk’ level, which I thought was really quite interesting. She phrased it…like she was disappointed to receive the result…and I thought, ‘oh, that’s really interesting to think about that’ because…there is no ‘lower than average risk’ level. So really you are getting the best mark if you want to put it that way.”*

(Participant 8)

Regarding facilitating risk communication on a large scale, educational videos aimed at enhancing risk literacy were suggested as one potential improvement to the model used in the study reducing the time required to conduct one-to-one conversations with a genetic counselor. Interviewees involved in risk communication also reflected on the inherent differences between the current approach to breast screening and the future state, highlighting the required education and shift in thinking for both the public and healthcare professionals regarding breast screening if a revised model of breast screening were to be implemented.

*“The PERSPECTIVE study identified some patients who did not have traditional hereditary cancer risk factors… so I think it would require a bit of a shift in thinking…and some education for genetic counsellors around other risk factors for breast cancer…it’s just the whole idea that you might have someone in high risk screening who doesn’t have a very significant family history would require some consultation and some discussion around how to implement that.”*

(Participant 9)

## 4. Discussion

One of the goals of PERSPECTIVE I&I is to determine optimal approaches for implementing risk-stratified breast screening within the Canadian health system. This qualitative study facilitated an in-depth exploration of the potential barriers and facilitators to delivering multifactorial risk assessment that were identified by our previous study [20]. To date, there has been only one other study, BC-Predict in the NHS Breast Screening Program in England, which evaluated barriers and facilitators of risk-stratified breast screening from the perspective of those delivering it on a large scale [22]. The findings from these two studies provide complementary evidence on distinct aspects of the risk-stratified screening pathway that is fundamental to decision-makers who are considering a shift to risk-based screening.

Our analysis identified five primary themes: (1) barriers and facilitators to participant adoption of multifactorial risk assessment, (2) barriers and facilitators to completion of the risk factor questionnaire, (3) additional resources required to implement multifactorial risk assessment, (4) the need for a person-centered approach, and (5) and risk literacy. Overall, the delivery of multifactorial risk assessment and risk communication was successful with nearly 4000 participants taking part despite recruitment occurring during the height of the COVID-19 pandemic. This highlights the effectiveness of a multi-pronged approach to recruitment, including mailed letters in Ontario (commonly used by organized screening programs to correspond with the screen-eligible population) and traditional media campaigns in Quebec, which may be an effective tool for promoting awareness and recruitment in future risk-stratified screening programs. Diverse strategies for driving screening participation in specific populations and geographies are already used by screening programs in Canada [33]; thus, programs can apply existing knowledge and processes to tailor recruitment strategies for risk-stratified screening.

While smooth operations were reported in many areas, several critical barriers were identified which require careful consideration as large-scale risk-stratified screening programs are developed. One of the central findings of our previous study was that participants appeared to encounter considerable difficulty completing the risk factor questionnaire [20]. This was confirmed by the findings of this study. Arguably, the largest challenge of delivering multifactorial risk assessment was the resource-intensive nature of collecting comprehensive and accurate risk factor information. Study personnel identified that the large amount and complexity of the risk factor information required was burdensome for participants, and there were functionality issues with the risk factor questionnaire web platform. Additional work is needed to optimize the process of collecting risk factor information before it can be executed on a population scale. Of critical importance will be identifying the minimum set of risk factors required to maximize both the accuracy of risk prediction and participation in the eligible population. Particularly challenging was the collection of first- and second-degree family history of breast, ovarian, pancreatic, and prostate cancer. Obtaining this information often requires participants to contact their relatives, which may have led individuals to leave some fields blank or pause the questionnaire to obtain this information, which may delay or prevent return of the completed questionnaire. Given these challenges, the impact on risk model performance of collecting only a first-degree family history should be investigated. Other studies of risk-stratified breast screening, such as BC-Predict [34], PROCAS [19], and WISDOM [14], have collected only first-degree family history, and a study is also underway to evaluate the predictive ability of BOADICEA using risk factor data from PERSPECTIVE I&I. Lastly, a robust web tool must be available for entry of risk factor information to ensure high-quality risk factor data can be collected. Notably, many of the validated breast cancer risk models, initially designed for clinical and research use, have released patient and public tools that could be used by screening programs.

This study also emphasized the need for embedding a person-centered approach that considers diverse lived experiences, the social determinants of health, risk levels, and individual preferences in processes for risk assessment and communication; that is, the personalized aspect of personalized screening must not be limited to just the clinical recommendations for screening and primary prevention. Our finding that varying preferences were held by participants for how risk level information is communicated, particularly according to different risk levels, is consistent with previous studies. For example, two European studies demonstrated that a high percentage of women felt that risk communication via a letter was acceptable for a low- or average-risk result, while more preferred that risk notification be over the telephone or face-to-face for a risk result that was above-average risk [17,35].

One of the major points which crosscut several themes was concern about the resources required to deliver risk-stratified screening in a constrained health system, particularly when scaled to the population level. As discussed above, significant resources were required to facilitate the multifactorial risk assessment, and personnel also discussed resource impacts on breast screening sites and for health care providers to facilitate risk communication and genetic counseling, where required. This is consistent with the concerns reported by health care providers in the BC-Predict study [22], as well as several other studies that examined perspectives from screening program administrators and health care providers regarding hypothetical future risk-stratified screening programs [36,37,38,39,40].

This study had many strengths. It is the first North American study to report on potential barriers and facilitators to delivering multifactorial breast cancer risk assessment and risk communication based on the experiences of personnel delivering these components of risk-stratified breast screening within an organized screening setting. Other strengths include the ability to provide rich insight into barriers and facilitators identified in our earlier quantitative work, the 100% response rate, and use of an interviewer and primary analyst with no prior involvement in the study which eliminated preconceived assumptions or biases in data collection and analysis. Several potential limitations should also be noted. Many of our findings reflect the study personnel’s reporting of study participants’ experiences. As they were not reported by participants directly, they may not accurately reflect participant perspectives. Additionally, most PERSPECTIVE I&I study participants were white, highly educated, and employed [22] and were further described in this study as “health motivated”. It is therefore likely that the findings were affected by healthy user bias, as personnel would not be able to comprehensively report on challenges unique to underrepresented groups. It is also possible that the data collected through the focus group were affected by social desirability bias in the discourse; however, this was mitigated by the methodology, including triangulation with findings from our quantitative study and collection of some data through interviews. Lastly, not all the findings may be generalizable to other health systems. Nonetheless, many may be transferable to other jurisdictions with organized breast screening programs.

Our study provides novel, instrumental knowledge on key aspects of the delivery of multifactorial risk assessment with polygenic risk scores in the general population, including recruitment, collection of risk factor information, risk estimation, and risk communication. While risk-stratified breast screening remains a promising strategy to overcome the limitations of age-based screening, additional evidence is still required on its clinical efficacy and the feasibility of population-based implementation. Our qualitative study identified many key barriers and facilitators and confirmed that significant efforts will be required to design robust, but less resource-intensive, processes for collecting, analyzing, and communicating risk factor information to permit population-scale implementation. This evidence can be used to inform the implementation of personalized breast screening on a larger scale in both Canada and other jurisdictions who are planning for a shift to a risk-based model of screening. Future phases of the PERSPECTIVE I&I study will provide further evidence on participants’ attitudes, psychological and emotional impacts, risk communication preferences, and satisfaction, along with screening and prevention behaviors and outcomes.

## Data Availability

Parts of the material underlying this article are based on data and information provided by Ontario Health (Cancer Care Ontario). Ontario Health is prohibited from making the data used in this research publicly accessible if they include potentially identifiable personal health information and/or personal information as defined in Ontario law, specifically the Personal Health Information Protection Act (PHIPA) and the Freedom of Information and Protection of Privacy Act (FIPPA). Upon request, data de-identified to a level suitable for public release may be provided.

## Supplementary Materials

The following supporting information can be downloaded at https://www.mdpi.com/article/10.3390/curroncol32030155/s1, File S1: Focus group and interview topic guides.
