# Supplementary material for: Barriers and Facilitators to Delivering Multifactorial Risk Assessment and Communication for Personalized Breast Cancer Screening: A Qualitative Study Exploring Implementation in Canada
Source: Curr Oncol. 2025 Mar 10;32(3):155. doi: 10.3390/curroncol32030155 (PMC11941251; doi:10.3390/curroncol32030155)
Supplement: Supplementary file 1 [file curroncol-32-00155-s001.zip › curroncol-3440078-supplementary.pdf]

PERSPECTIVE
INTEGRATION & IMPLEMENTATION

Topic Guide – Recruitment, Data Collection & Validation

Recruitment

First, we are going to talk about your experiences with the processes surrounding women being recruited to the study.

1. Bearing in mind that different approaches were employed to recruit women into the study in Ontario and Quebec: What aspects of these approaches would you say worked well? What didn't work well?

2. What factors do you think influenced the effectiveness of the various approaches to recruitment?

3. Thinking beyond the study, could you see the same approaches being used successfully to invite women to participate in risk-based breast screening provincially? Why/why not?

Data collection

Now we are going to talk about your experiences with the initial data collection processes for the information used to estimate women's breast cancer risk level.

4. Did women contact you for any reason relating to completion of the entry questionnaire?
    - If so, did their reasons for contacting the study team differ depending on whether they were completing their questionnaire online or via the phone or on paper?

5. Did women contact you for any reason relating to providing saliva samples?
    - Did women encounter challenges with providing saliva samples?
    - If so, how did you deal with these challenges?

6. Thinking about your experience with managing the flow of collected saliva samples to the lab, what aspects of this process worked well?
    - Did you experience any challenges with this or related processes?

7. Thinking about your experience with retrieving mammogram reports, what aspects of this process worked well?
    - Did you experience any challenges with retrieving mammogram reports?
    - Did you experience any challenges with abstracting mammographic density from the reports?

8. Could you see the same approaches (as those used in the study) being used successfully to collect information in a risk-based breast screening program provincially? Why/why not**?**
    - Was there anything that you feel could have improved the process of data validation?
    - What do you think would be the ideal approach to validating information used to estimate breast cancer risk?

Data validation

Now we are going to move on to talk about your experiences with validating women's questionnaire data, for example in the case of missing or unusual risk factor information.

9. Can you describe the steps involved in the process of data validation in your own words?
    - How would you validate missing or unusual risk factor information?
    - How long did these conversations usually last? What factors influenced how long the conversations lasted?
    - Were there ever issues that could not be resolved/ what did you do in cases of unresolved issues?
    - Did you find you had to adapt your process over the course of the data collection period? When/why/why not?
    - Did you work together as a team to complete any aspects of the data validation?

10. Did you have issues with getting in contact with the participants?
    - Did you experience any language barriers?
    - Did you experience any technical or other issues?
    - What are your views on completing the data validation conversations via telephone?

11. What parts or aspects of the questionnaire did women find most challenging? Why do you think that was?
    - Reasons for follow-up often related to missing information relating to family history, whether a breast cancer was unilateral or bilateral, missing risk factor information, missing year of birth or age at diagnosis – why do you think these items were the most frequently missed and/or requiring verification?

12. Did participants ever voice concerns relating to data validation to you?
    - Did participants ever voice any perceived benefits relating to data validation?
    - Did you feel that study participants were open to sharing personal and/or sensitive information with you?

13. How would you describe your overall experience of your role in completing data validation for the study?
    - What were the most challenging aspects of your role in the data validation process?
    - What were the most enjoyable aspects of your role in the data validation process?
    - Did you feel confident in your ability to have these conversations with women, particularly when thinking about topics often viewed as sensitive, such as alcohol consumption, pregnancy loss and cancer?

14. Could you see the same approaches (as those used in the study) being used successfully to validate information in a risk-based breast screening program provincially? Why/why not**?**
    - Was there anything that you feel could have improved the process of data validation?
    - What do you think would be the ideal approach to validating information used to estimate breast cancer risk?

15. Is there anything I haven't covered that you thought we would discuss?

<u>Topic Guide – Risk Communication</u>

1. In your own words can you please describe your role in this part of the study?
    - What are the goals/aims of genetic counselling? Did this differ within the context of your role in the study?
    - How was breast cancer risk communicated to women deemed to be at 'high risk' in the study? Did you approach this the same way for everyone?
    - How long did these conversations usually last? What factors influenced how long the conversations lasted?

2. How would you describe your overall experience of your role in the study?
    - What were the most challenging aspects of your role as genetic counsellor/clinical advisor/study nurse?
    - What were the most enjoyable aspects of your role as genetic counsellor/clinical advisor/study nurse?
    - Did you work together as a team to complete any aspects of your role?

3. What were the main challenges *you* encountered relating to your role?
    - Did you have issues with getting in contact with women? Language barriers? Technical issues?
    - What are your views on completing the conversations via telephone?

4. Did you feel that women were open to share personal and/or sensitive information with you?

5. Did women voice concerns relating to genetic counselling/risk communication to you?
    - *If yes,* what were some of these concerns you remember?

6. Were there ever issues that could not be resolved/ what did you do in cases of unresolved issues?

7. Did you find you had to adapt your process over the course of the study period? When/why/why not?

8. Was there anything that could have made your role/the process easier?

9. Did you feel adequately prepared/how confident were you in your ability to have conversations with women as part of the study?

10. Thinking beyond the study, could you see the same approaches being used successfully to communicate with women estimated to be at high-risk of breast cancer as part of a risk-based breast screening program provincially? Why/why not**?**
    - What do you think would be the ideal approach to providing genetic counselling/communicating breast cancer risk to women at high risk?

11. Is there anything I haven't covered that you thought we would discuss?
